# The accordion technique did not improve bone healing in a mouse model of distraction osteogenesis

**DOI:** 10.1038/s41598-024-71335-0

**Published:** 2024-10-18

**Authors:** David T. Bertrand, Ruisen Fu, Kyle Kavaseri, Isabelle Villemure, Frank Rauch, Reggie Hamdy, Haisheng Yang, Bettina M. Willie

**Affiliations:** 1 Research Centre, Shriners Hospital for Children-Canada, Montreal, Canada; 2 Faculty of Dental Medicine and Oral Health Sciences, McGill University, Montreal, Canada; 3 Department of Biomedical Engineering, Beijing University of Technology, Beijing, China; 4 Department of Mechanical Engineering, Polytechnique Montréal, Montreal, Canada; 5 Department of Pediatrics, McGill University, Montreal, Canada; 6 Department of Pediatric Surgery, McGill University, Montreal, Canada

**Keywords:** Bone healing, Mechanobiology, Accordion technique, Distraction osteogenesis, Finite element modeling, Bone, Preclinical research, Fracture repair

## Abstract

Distraction osteogenesis (DO) is a valuable surgical method for limb lengthening and bone defect correction, but its lengthy consolidation phase presents challenges. The accordion technique (AT), involving compression and distraction of bone segments, has shown potential for enhancing healing. This study aimed to investigate the effectiveness of the AT conducted at three different time points (distraction phase, early consolidation phase, or late consolidation phase) compared to conventional DO in a mouse osteotomy model. Healing was evaluated using in vivo microCT, histology, and computational modeling. Results showed that bridging frequency, BV, and callus tissue composition were similar between conventional DO and late consolidation AT. In contrast, distraction phase AT led to delayed healing at day 15 with a 72% reduction in BV compared to DO, but no significant differences by the endpoint. Early consolidation AT showed significantly impaired healing compared to DO, with only 29% of mice achieving bony bridging, and significantly reduced bone marrow area of the endpoint callus. In silico modeling was generally predictive of in vivo findings and suggested that application of the AT during early consolidation results in destruction of newly-formed vascular tissue. Overall, no benefit was observed for the AT compared to conventional DO with the parameters employed in this study.

## Introduction

Distraction osteogenesis is a surgical technique involving the controlled, gradual distraction of bone segments. Following a procedure during which a distraction-capable fixator device is attached to the target bone and a corticotomy or osteotomy is performed, distraction osteogenesis consists of 3 main phases^1^. First, there is the post-surgical latency period, during which time the bone is left undisturbed, and the early stages of bone healing, such as hematoma formation and recruitment of mesenchymal stem cells, are allowed to take place^2^. In humans, a timeline of 5–7 days is recommended for this phase, as was originally suggested and enacted by Ilizarov^1^. Following the latency period is the distraction phase, in which the bone segments are slowly pulled apart, typically carried out at the recommended rate of 1mm/day and frequency of 2–4 times/day^1^. During this phase, the callus initially formed during the latency period is elongated and partially resorbed, and a fibrous interzone is formed in the gap containing collagen fibres oriented parallelly to the direction of distraction^3,4^. Finally, once the required distraction length has been achieved, the consolidation phase begins. This involves a long period of stabilization, during which the distraction fixator device must remain in place. Once sufficient healing has occurred, and the quality of formed regenerate is deemed sufficient, the device is finally removed in a follow-up surgery^5^.

While DO is widely considered a safe and effective procedure, there is a significant impact on patient quality of life, particularly due to the length of the consolidation phase. Healing during this stage of DO can last up to several months, and, as a result, carries with it a psychological strain for the patient, as well as increased risk of infection and osteopenia^6–8^. In recent years, clinicians have begun, in many cases, to move away from Ilizarov external ring fixators toward less invasive options such as motorized intramedullary nails and integrated limb lengthening^9,10^. Nevertheless, there is great interest in enhancing healing during the consolidation phase in an effort to improve patient outcomes and quality of life during treatment.

Previous research has shown that alternating distractive and compressive forces applied during fracture healing may accelerate and improve outcomes^11^. In the case of DO, this principle has been applied in the past in what is known as the accordion technique (AT), which involves the incorporation of alternating compression and distraction into the DO protocol. In several clinical cases where healing outcomes following DO were inadequate, the AT was applied, leading to successful bridging in several patients^12–14^. However, while reactive application of the AT following poor healing has yielded promising results, this technique has not yet been clinically employed for the purpose of proactively accelerating regeneration during the consolidation phase. Previous studies examining alternating compression and distraction during DO in animal mandibles have shown promise^15–17^, and limited preclinical and clinical evidence has suggested that the incorporation of compression into a DO protocol can improve healing, either when done during the distraction phase^13,18^ or consolidation phase^19^.However, the feasibility, precise optimal timing, and physiological processes underpinning application of the AT in long bone DO remain unclear.

**Fig. 1:**
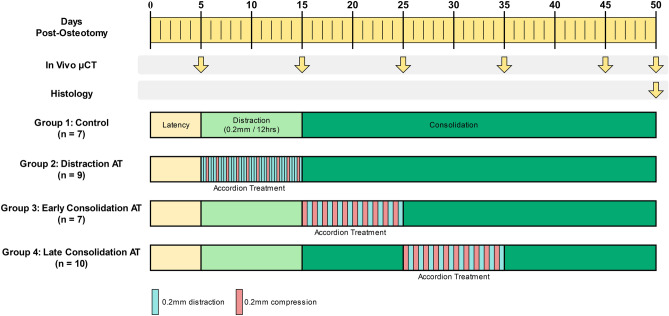
Timeline of distraction osteogenesis (DO) and accordion technique (AT) procedures following osteotomy surgery at day 0. All groups underwent a standard distraction phase of 0.2 mm/12 h, except Group 2, which underwent the AT during the distraction phase. Groups 3 and 4 underwent the AT during the consolidation phase at days 15–25 and 25–35 post-osteotomy, respectively. Timing of the in vivo microCT scans and histology analysis is indicated by arrows.

**Fig. 2:**
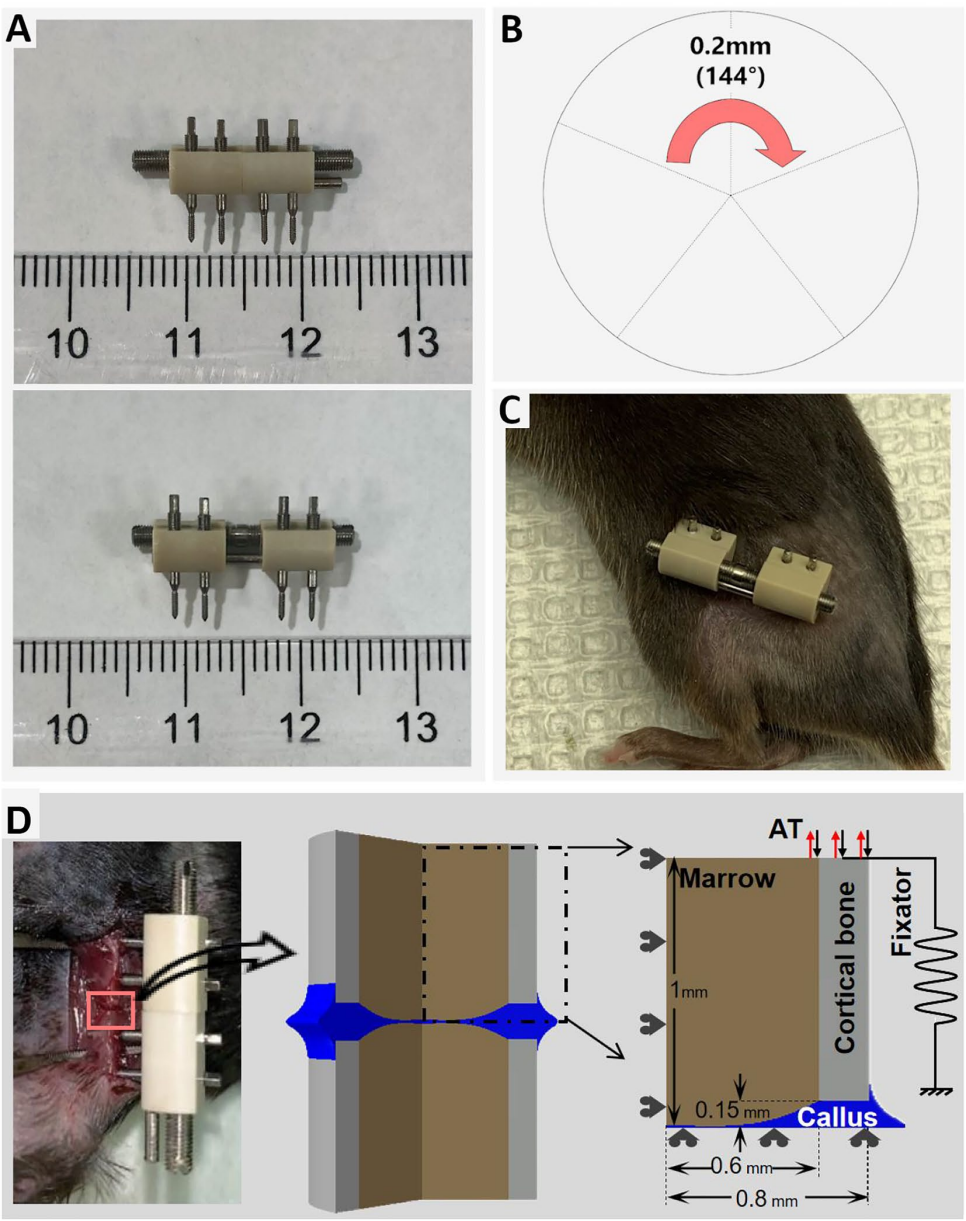
The fixator/distractor system used for distraction osteogenesis and the accordion technique in our experiments. (**A**) During the surgical procedure, MouseDis fixators (RISystem, Switzerland) were mounted onto the left femur prior to the creation of an osteotomy (upper) image. Following osteotomy surgery, distraction and compression was performed resulting in a 4 mm gap (lower image). (**B**) Distraction or compression could be performed simply by turning the threaded bar clockwise or counterclockwise, respectively. (**C**) Throughout the healing period, the external MouseDis fixator device can be manipulated on the mouse. (**D**) A two-dimensional axisymmetric finite element model of the osteotomized site was created according to experimental measurements of the femoral geometry. The initial osteotomy gap was 0.3 mm. The inner and outer diameters of the cortical bone are 1.2 and 1.6 mm, respectively. The fixator was modeled as a linear spring.

The primary objectives of this study were as follows: (1) To determine how alternating compression and distraction applied in a murine femoral osteotomy model would affect healing outcomes, and (2) to predict the progression of healing at the distraction site using finite element modeling and mechanobiological simulations. We hypothesized that application of the accordion technique would lead to improved and accelerated healing throughout the consolidation phase when compared with conventional DO (Fig. 1) in a murine femoral osteotomy model (Fig. 2).

## Results

### Healing was similar following conventional distraction osteogenesis and the accordion technique applied during distraction or late consolidation

**Fig. 3:**
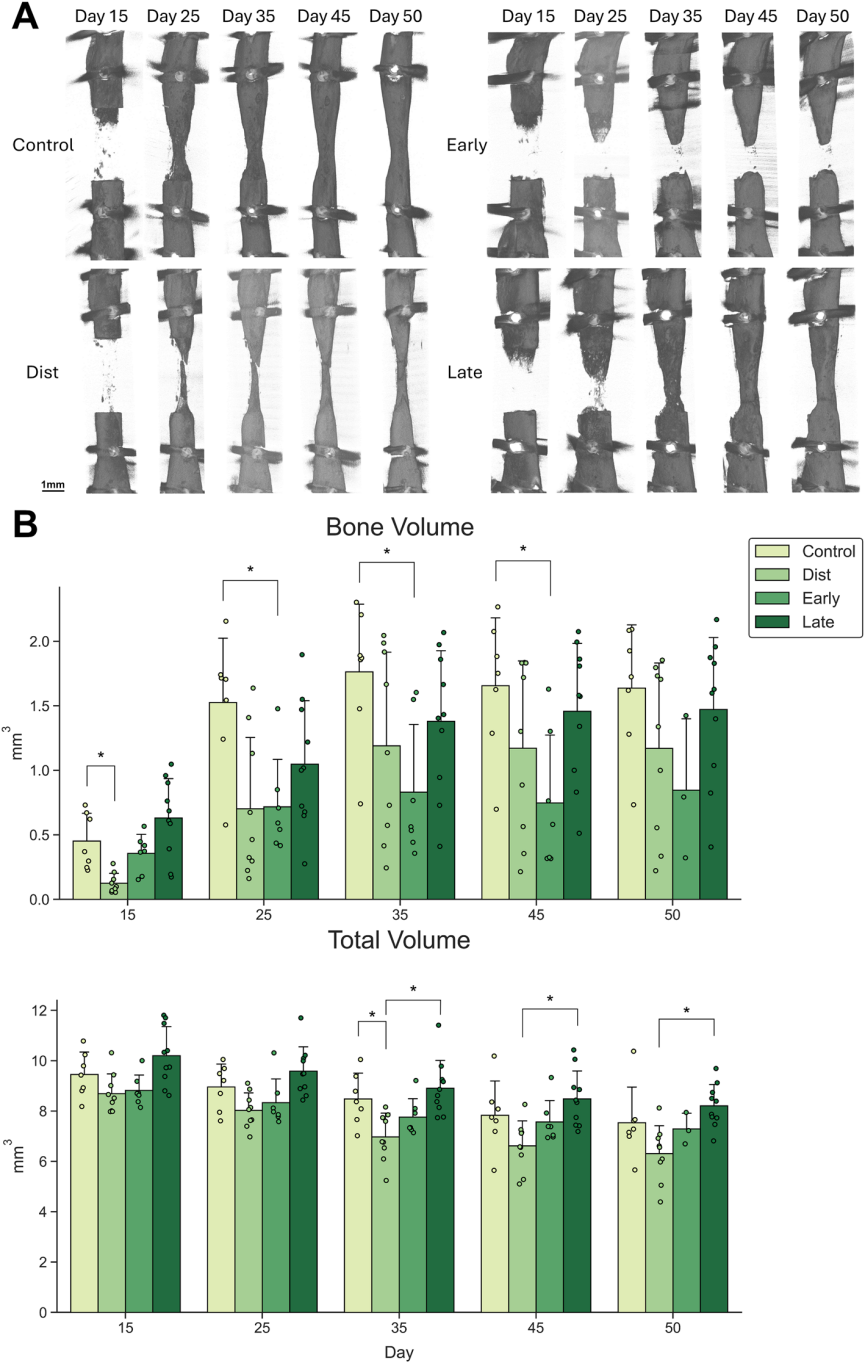
(**A**) Representative in vivo microCT images from each experimental group taken at days 15, 25, 35, 45, and 50 post-osteotomy. (**B**) In vivo microCT outcomes assessed at various time points. Bone volume (BV) was similar between Group 1 (DO), Group 2 (distraction AT) and Group 4 (late consolidation AT) throughout the healing period. In contrast, BV was significantly increased in the DO group compared to Group 3 (early consolidation AT) mice beginning at day 25, indicating that healing was markedly impaired in the early consolidation AT mice. Total volume (TV) decreased over time in all groups, as any newly formed bone tissue took its final shape. Values are reported as Mean ± SD. ANOVA: (a) Treatment group, (b) microCT time point, (c) interaction, asterisk indicates significant difference based on Tukey–Kramer post-hoc test. Significance for all tests was set at p < 0.05.

I*n vivo* microCT scans to evaluate healing progression revealed no significant differences in mineralized callus volume (BV) between the DO and either late consolidation AT group or the distraction AT group (Fig. 3A) as of day 25 onward. There was no difference measured at any of the time points between DO and late consolidation AT groups, indicating similar progression of mineralized tissue formation throughout the healing period. Interestingly, BV was significantly decreased by 72% in mice where the AT was performed during the distraction phase compared to conventional DO mice at day 15 post-surgery. This would indicate a delay in the early stages of tissue mineralization in the distraction AT group at day 15, which is the last day of the distraction phase. However, no difference in BV between these groups was observed at later time points. The distraction AT group was able to recover after initially having a lower BV at days 15 (72%) and 25 (54%) than the DO group, the latter of which was not statistically different.

Regarding healing progression within each group over time, we observed a significant increase in BV for all groups between days 15 and 35, while BV did not increase significantly in any group between days 35 and 50. In fact, BV significantly decreased between days 35 and 50 in the DO group, likely due to reconstitution of the medullary canal between this time points. This would indicate that the bulk of mineralized tissue formation occurred within the first 35 days post-osteotomy, or within the first 20 days of the consolidation phase.

By the endpoint (day 50), 85% of mice undergoing conventional DO had achieved bony bridging as assessed by microCT scans. Bridging score was 70% for the late consolidation AT group and 55% for the distraction AT group (Table 1). Comparisons between groups using Fisher’s Exact test revealed that bridging frequency of the distraction AT and late consolidation AT groups did not significantly deviate from the DO group.

**Table 1:**
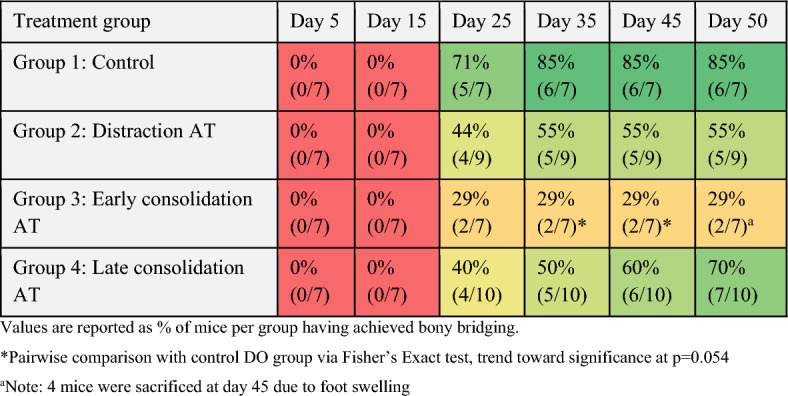
Bony bridging score assessed by microCT scans at all time points.

Values are reported as % of mice per group having achieved bony bridging.

^a^4 mice were sacrificed at day 45 due to foot swelling.

ANOVA revealed a significant effect of treatment group, microCT time point, and an interaction on total callus volume (TV) (Fig. 3B). A significant decrease in TV was measured across all four experimental groups (DO, dist AT, early AT, and late AT) over the course of the healing period (from day 15 to day 50). This decrease in TV within each treatment group between day 15 and day 50 was most pronounced in the distraction AT group: (20% DO, 27% dist AT, 17% early AT, 19% late AT).

This unequal decrease in TV across all treatment groups led to significant differences notably, at day 35; the distraction AT group showed significantly decreased TV compared to the DO group, indicating a volumetrically smaller distraction callus. However, this difference was not observed at the endpoint (day 50).

**Fig. 4:**
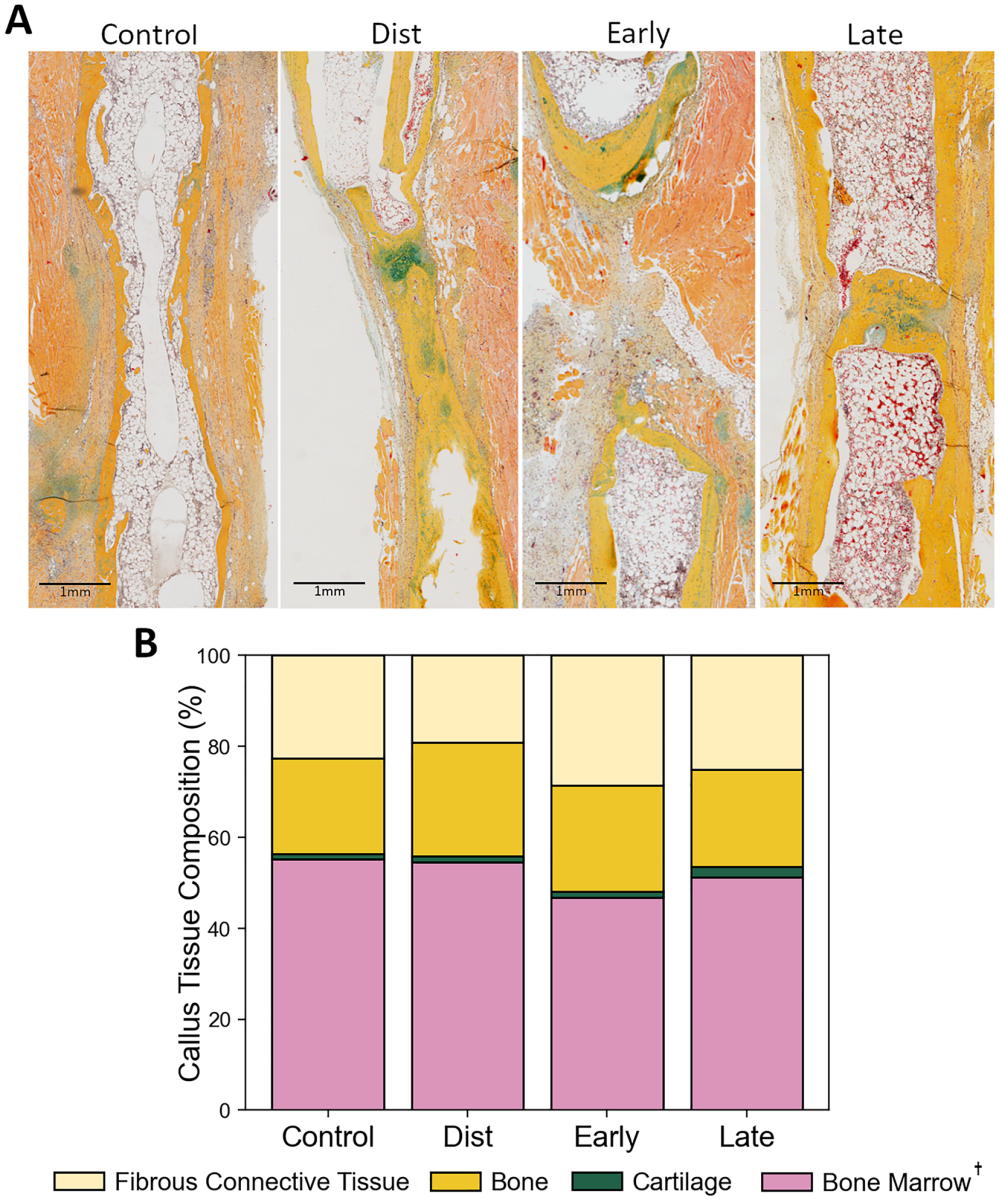
(**A**) Representative histological images of distraction calluses from the day 50 experimental endpoint. Femurs were embedded in paraffin, sectioned, and stained with Movat Pentachrome, which allows for visual differentiation between fibrous connective tissue (grayish), cortical bone (yellow), cartilage (blue-green), and bone marrow (reddish pink). (**B**) The area fraction of bone, cartilage, fibrous connective tissue, and bone marrow present in the fracture callus at day 50 measured from the Movat Pentachrome-stained histological sections. Bone marrow was significantly decreased in the early consolidation AT group, indicating less advanced healing outcomes. Dagger: between-subject effect of Treatment group, ANOVA, p < 0.05.

Histological analysis via Movat Pentachrome staining corroborated many of our aforementioned microCT findings. While the incidence of bridging was similar between the DO, distraction AT and late consolidation AT groups, qualitative histological assessment revealed that mice in the DO group more often achieved an ideal healing outcome (full reconstitution of the cortical shell, medullary canal, and bone marrow) (Fig. 4A). Quantitative histological analysis of callus tissue composition revealed no significant difference in the amount of bone, cartilage, bone marrow or fibrous connective tissue between the DO, distraction AT and late consolidation AT groups (Fig. 4B). However, interestingly, while total callus volume was similar between the DO and late AT groups, it was significantly decreased in distraction AT mice compared to DO mice (Table 2). This was largely due to the amount of fibrous connective tissue measured in the callus compared to the other treatment groups.

**Table 2:** Total callus area, bone area, cartilage area, fibrous connective tissue area, and bone marrow area for each treatment group, mean ± SD.

	Control	Dist AT	Early AT	Late AT
Bone marrow area (mm^2^)^†^	3.159 ± 0.472	2.168 ± 1.244	1.733 ± 0.290*	2.927 ± 0.771
Bone area (mm^2^)	1.204 ± 0.435	0.993 ± 0.600	0.867 ± 0.072	1.214 ± 0.366
Cartilage area (mm^2^)	0.060 ± 0.063	0.052 ± 0.054	0.047 ± 0.050	0.138 ± 0.195
Fibrous connective tissue area (mm^2^)	1.293 ± 1.078	0.760 ± 0.719	1.060 ± 0.560	1.436 ± 0.635
Total callus area (mm^2^)	5.715 ± 1.068	3.973 ± 0.481*	3.707 ± 0.622*	5.714 ± 0.988

^†^Between-subject effect of Treatment group, ANOVA, p < 0.05.

*Independent t-tests between AT Treatment groups and control DO, p < 0.05.

### Healing following conventional DO was superior to the accordion technique applied during early consolidation

There was a significant effect of treatment group and microCT time point on BV. Paired comparisons carried out via Tukey post-hoc testing revealed that BV was significantly higher at days 25, 35 and 45 post-surgery in mice undergoing conventional DO compared to mice subjected to the AT during early consolidation (Fig. 3A). Mice from the early consolidation AT group therefore exhibited delayed and impaired mineralized tissue formation throughout the healing period compared to the DO group.

The early consolidation AT group had the lowest bridging score at 29%. It must be noted that four mice from this experimental group required euthanasia at day 45 due to swelling of the foot. However, only two mice from this entire group showed any signs of bridging by this time point. Bridging of mineralized tissue occurred far more frequently by day 50 post-surgery in the conventional DO group (85%) than the AT applied during early consolidation (29%) (Table 1). Statistical analysis using Fisher’s Exact test revealed that this difference trended toward significance (p = 0.054), indicating impaired healing in the early consolidation AT group compared to the conventional DO group. Both quantitative and qualitative histological analysis of callus tissue composition revealed superior healing outcomes in mice undergoing conventional DO when compared with early consolidation AT mice (Fig. 4). Qualitatively, the callus in mice undergoing the AT early in the consolidation phase generally showed less advanced healing, with much lower rates of mineralized tissue bridging and cortical capping of the bone segments, suggesting the onset of nonunion. Quantitative analysis of callus tissue composition showed significantly increased bone marrow area in DO mice compared to early consolidation AT mice, indicating an improved and more advanced healing outcome.

### In silico modeling of healing patterns under various AT parameters were predictive of in vivo results and related to the mechanical environments within the distraction gap

**Fig. 5:**
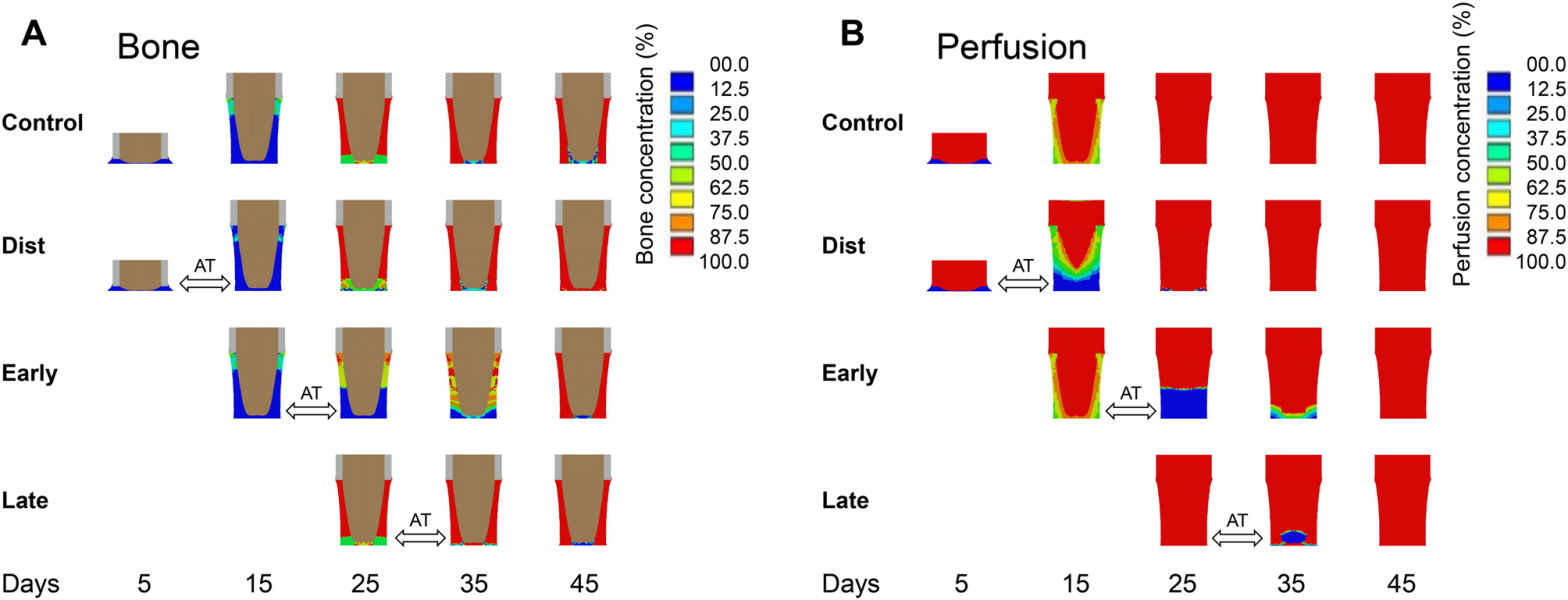
The tissue distribution (**A**) and blood perfusion concentrations (**B**) in the distraction gap for conventional DO and accordion groups, as predicted by in silico modeling. Tissue concentration in (**A**) refers to the density of mineralized tissue, while in (**B**) it refers to vascular density.

**Fig. 6:**
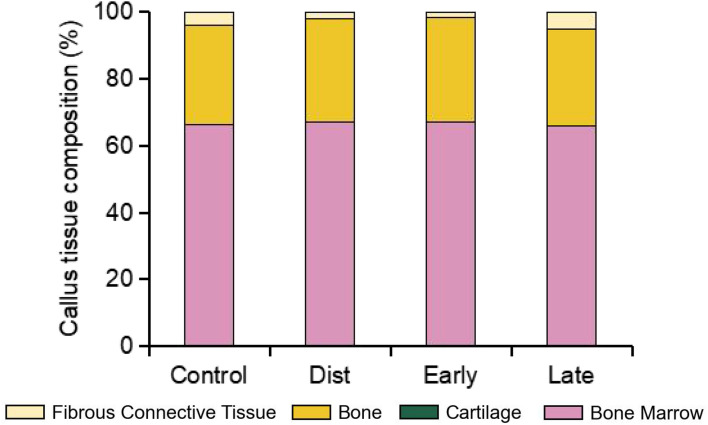
Callus tissue composition predicted by in silico modeling at day 50. The model predicted modest differences in bone and fibrous connective tissue across the different experimental groups, but uniformly high levels of bone marrow from a reconstituted medullary canal and no cartilage.

Computer models predicted the overall progression of tissue regeneration over the course of healing in mice undergoing DO and AT procedures (Figs. 5, 6). For conventional DO, according to the model’s predictions, the initial bone formation occurred during the distraction phase in the longitudinal direction, primarily via intramembranous ossification (Fig. 5). During the consolidation phase, from days 15–25, bone grew rapidly and bony bridging occurred, after which the callus eventually became fully composed of newly formed bone tissue. Similar phenomena were observed when the AT was applied during late consolidation. When the AT was applied during the distraction phase, tissue formation appeared to be significantly delayed by day 15, but healing and bony bridging at subsequent time points was consistent with conventional DO (Fig. 5). Finally, when the AT was applied during early consolidation, markedly delayed healing was observed compared to the conventional DO group.

**Fig. 7:**
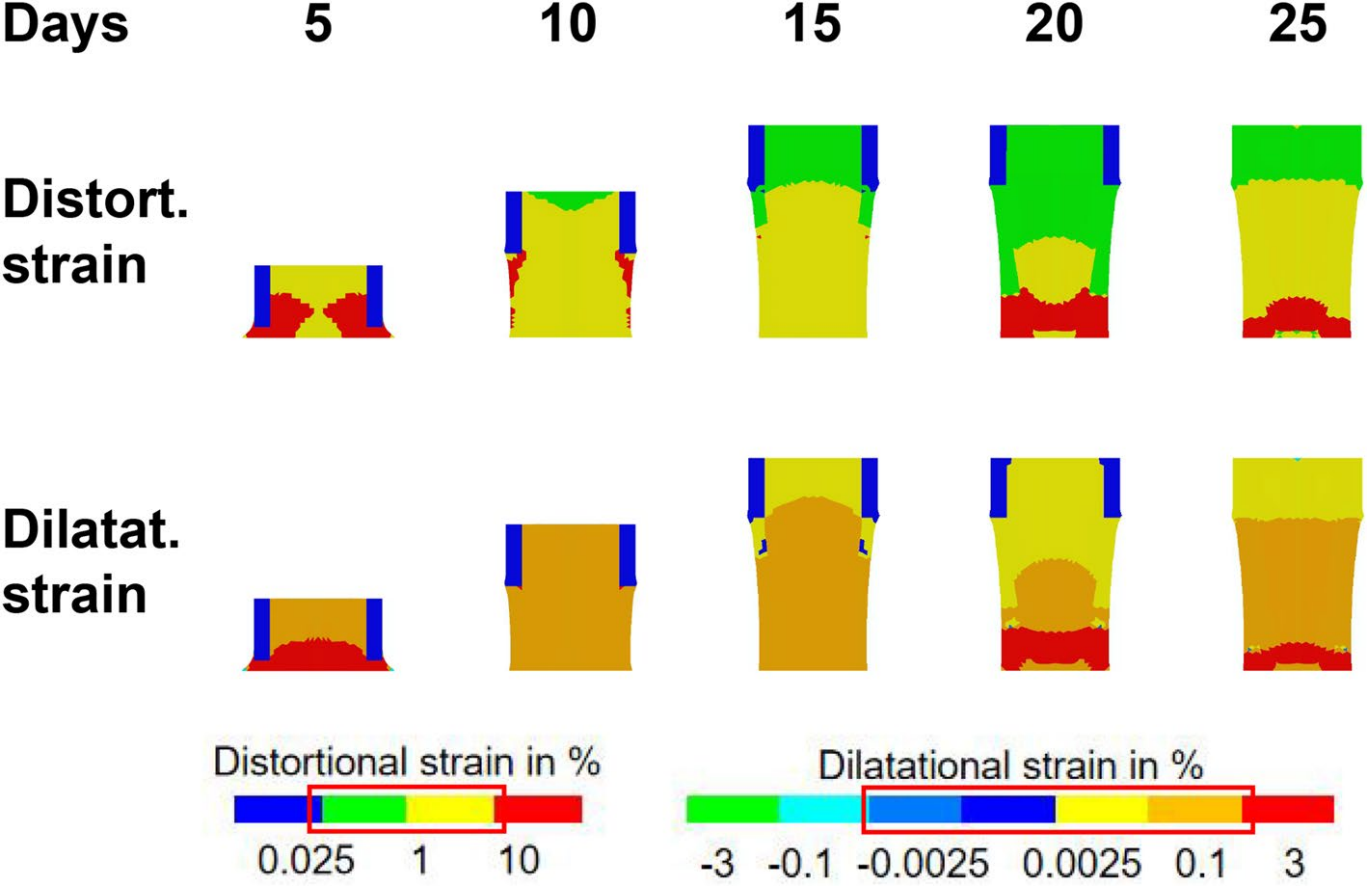
Dilatational and distortional strain states generated within the distraction gap when the AT is applied at various time points throughout the process of DO. The red box corresponds to mechanical stimulation thresholds for bone formation in a mechano-regulatory tissue differentiation model.

In terms of the mechanical environment produced by the AT rate of 0.2 mm/12 h, the magnitude of interfragmentary strain (IFS) was high (ε > 3 and γ > 10) in the distraction gap throughout the first half of the distraction period (days 5–10) (Fig. 4). Then, IFS was reduced to a far lower magnitude (0.01 < ε < 3 and γ < 10), with the continuous extension of the distraction gap from days 10 to 15 (Fig. 7). However, IFS generated by AT procedures gradually increased to a higher level (ε > 3 and γ > 10) with the growth of new bone tissue, when the AT was applied during the consolidation phase (day 15–25) (Fig. 7).

## Discussion

Our first hypothesis, which was that the AT would enhance healing outcomes compared to mice undergoing conventional DO, was not supported by our results. In fact, for the distraction and late consolidation AT groups, no significant benefit of the application of the AT was observed across any examined metric compared to conventional DO. In vivo microCT assessment revealed no improvement in the rate of bony bridging and no consequential differences in mineralized tissue volume (BV) throughout the healing period in the AT groups compared to the conventional DO group. Histological findings also corroborated these results, showing similar callus tissue compositions within the distraction gap between the conventional DO, distraction, and late consolidation AT groups. These results were surprising, particularly within the late consolidation AT group, as previous studies have suggested positive outcomes of applying compressive forces to long bones undergoing DO during this approximate time period. One such study showed that interfragmentary movement during the consolidation phase of DO could enhance maturation of the distraction callus in sheep^20^. In this case, differences in interfragmentary movement magnitudes were the result of varying fixation stiffnesses and not by directly applying compression and distraction at specific time points, as was done in the present study. Additionally, in a recent study, Xu et al*.* reported markedly accelerated healing during the consolidation phase in rat tibias, with significantly increased BV/TV and BMD compared to controls occurring when compression of the distraction gap was started 2 weeks following the end of the distraction phase.^19^ Liu et al*.* also showed similar findings by employing the same protocol in rat femurs, including an increase to BV/TV, BMD, and energy to failure when bones were subjected to the AT instead of conventional DO.^21^ They also recently observed improved healing outcomes, such as increased callus BV, ultimate load, and energy to failure when the accordion technique was applied during the distraction phase with low amplitude (0.75 mm distraction, 0.25 mm compression at 12-h intervals).^18^ The differences in our results serve to underscore the likely importance of timing and distraction/compression parameters when attempting to incorporate compression into DO. Another possible reason for the discrepancy between our findings and the other aforementioned studies is that our study used mice instead of rats. It has been shown that the mechano-biological regulation of bone regeneration differs from mice and rats as well as other species^22,23^.

Our second hypothesis, which was that in silico modeling could predict in vivo healing outcomes of using the AT at various time points, was supported in that our in silico model was generally predictive of the outcomes of the in vivo experiment. Computer modeling revealed that, when the AT was applied during the distraction phase, a delay in the formation of mineralized tissue occurred until day 25 (Fig. 5). This was also observed in vivo, where a significant decrease in BV was observed at day 15, immediately post-distraction, when compared with the conventional DO group (Fig. 2A). Previous studies have shown that the bulk of angiogenesis supporting the subsequent formation of bone tissue occurs during the distraction phase^24,25^. It is thus plausible that application of the AT during this time point could have inhibited angiogenesis, resulting in delayed ossification, as predicted by our model (Fig. 5B). Furthermore, in silico modeling of the distraction gap showed that application of the AT during the first half of the distraction phase (between days 5 and 10) resulted in destructive magnitudes of dilatational and distortional strain (Fig. 7)^26^. However, this deleterious effect may have been mitigated by the lower strains engendered by the AT throughout the later parts of the distraction phase (days 10 to 15). By the day 50 endpoint, both in our in silico model and our in vivo experiments, tissue formation and rate of bony bridging were not markedly different for the distraction AT and late consolidation AT groups when compared with conventional DO. This would indicate that the distraction AT group was able to recover from its initial delay in ossification, while application of the AT during late consolidation did not result in notably enhanced or delayed healing. This could be explained by the majority of woven bone formation and bony bridging occurring prior to day 25, limiting the potential osteogenic benefits of any treatments applied from this time point onward.

For the early consolidation AT group, it was apparent across all measured experimental parameters that application of the AT immediately following the distraction period between days 15–25 resulted in significantly delayed healing. In vivo microCT analysis showed significantly reduced BV in the early consolidation AT group compared to conventional DO as of day 25, and the rate of bony bridging was reduced in these mice compared to conventional DO as well. Quantitative histological analysis supported these findings, revealing increased fibrous connective tissue and decreased bone marrow in calluses collected from the early consolidation AT group, indicating reduced bony bridging and subsequent reconstitution of the medullary canal. These findings were surprising, as previous studies which incorporated compression following DO in animal models did not exhibit such powerful inhibitory effects on the healing process at any of their measured time points^15–17,19,21^An in silico model we previously developed in sheep^27^ showed that the healing effect of the AT was strongly dependent on the application time, duration, and rate of the AT. Our previous model found that higher accordion rate for a longer duration resulted in delayed healing. Thus, these previous results and our current data further highlight the critical importance of carefully selected parameters for application of the accordion technique, as differences in protocol can evidently drastically impact DO healing outcomes.

Upon examination of the results from our own in silico modeling, it becomes clear that they were also predictive of the delay in healing caused by application of the AT during early consolidation. A delay in the formation of newly formed mineralized tissue was predicted (Fig. 5A), and this was also observed in our in vivo microCT results (Table 1; Fig. 3), as previously described. The in silico model suggests that this outcome is at least partially the result of destruction of newly formed vascular tissue caused by the alternating compression and distraction involved in the AT (Fig. 5B). This was further corroborated by dilatational and distortional strain predictions, indicating that application of the AT between days 20 and 25 results in supraphysiological and destructive magnitudes of strain (Fig. 7). However, it is notable that, while the model predicted an eventual delayed onset of bony bridging, most mice from the early consolidation AT group in our in vivo experiment simply produced a non-union and did not achieve full bridging by the endpoint at day 50. This may be because the in *silico* model did not consider the impact of tissue destruction, meaning that as long as conditions are suitable, tissue can still differentiate into bone. Therefore, the model can only predict delayed healing and cannot predict non-union.

In regular fracture healing, the mechanical environment created by compressive, tensile, and shear forces engendered during interfragmentary movements is critical in determining the composition of tissues formed within the callus. It is well known that there is an optimal window of interfragmentary movement which promotes favourable healing outcomes^28^, and it has also been shown that dynamization is beneficial to bone regeneration^29^. However, DO is a regenerative process with substantial differences to conventional fracture healing, with a far greater emphasis placed on factors such as tensile tissue strains and intramembranous ossification. At present, the relationship between mechanical stimulation and healing outcomes in DO remains under-researched and poorly understood. While the incorporation of compression into DO protocols has shown some promise in previous research, both the in vivo and in silico results of this study demonstrate the critical importance of optimizing timing and application parameters of the AT.

This study had some limitations. While the methods employed in this study allowed for in-depth quantification of tissue-level responses to the application of the AT during DO, we did not assess underlying molecular processes. However, we were able to investigate several mechanisms underlying our findings using in silico modeling. Furthermore, as with any in vivo study, our mouse experiments had limited ability to explore a large variety of different DO and AT parameters, such as fixation stiffness, distraction/compression rate, total length and frequency. We have previously observed, in both animal and computational studies, the critical importance of such parameters, especially in determining healing outcomes^30,31^. Thus, further in vivo and in silico experimentation is required to further explore the potential of the accordion technique.

## Conclusions

In summary, this study sought to determine how the application of alternating compression and distraction, otherwise known as the accordion technique, in a murine model of distraction osteogenesis affected healing. Our key findings were (1) that while the progression of healing following DO can differ greatly depending on the parameters employed, application of the AT during the distraction phase (days 5–15) and the late consolidation phase (days 25–35) did not significantly improve healing outcomes when compared to conventional DO. (2) We found that mice undergoing the AT during the early consolidation phase (days 15–25) exhibited significantly delayed healing outcomes. (3) In silico modeling revealed that the AT employed with alternating 12-h 0.2 mm compressions and distractions either during the distraction phase, early consolidation phase or late consolidation phase, can result in destructive magnitudes of distortional and dilatational strain when applied before day 10 and after day 15. Overall, our findings suggest that, while the incorporation of compressive manipulations has shown promise for both fracture and DO healing in various previous studies^11,13,32^, great care must be taken when selecting parameters for such manoeuvres. Additional research is required to establish optimal protocols for application of the AT; for instance, our in silico modeling would suggest that a future study employing alternating compression and distraction over a shorter 5-day window between days 10 and 15 post-osteotomy would lead to superior healing outcomes. Although anecdotal clinical evidence has shown promise when the AT was applied following a poor DO healing outcome, with the methodology employed in this study, we did not observe a benefit to proactive use of the AT to enhance healing during the consolidation phase.

## Methods

A total of 33 female *C57BL/6* mice (Jackson Laboratories, Bar Harbour, ME, USA) were received and acclimatized in the animal facility at the Shriner's Hospital for Children, housed 3 per cage with a 12-h alternating light/dark cycle. The mice were then randomized into 4 experimental groups: Group 1) conventional distraction osteogenesis (DO) (n = 7 mice), Group 2) accordion technique (AT) during the distraction phase (n = 9), Group 3) AT during the early consolidation phase, from day 15 to 25 (n = 7), and Group 4) AT during the late consolidation phase, from day 25 to 35 (n = 10) (Fig. 1). All animal use was approved by the Shriner's Facility Animal Care Committee (AUP 7821), and animal welfare was closely observed daily by the animal facility technicians. All experiments were performed in accordance with relevant guidelines and regulations. Reporting of this study was carried out in accordance with ARRIVE guidelines.

### Surgical procedure

Prior to surgery, mice were injected with slow-release buprenorphine, followed by clindamycin (common name: Baytril) at 45 mg/kg. The mice were anesthetized via inhaled isoflurane (1.5%-2% pure isoflurane oxygen mixture), and the area surrounding the left femur was shaved and sterilized. An incision was performed on the superficial dermal layer atop the left femur; the fascia lata was bluntly dissected, and the femur was exposed using a pair of forceps by separating the M. vastuslateralis and M. biceps femoris. A unilateral external fixator (MouseDis system, RISystems, Switzerland) (Fig. 2A) was mounted in the cranio-lateral direction by inserting four 0.45 mm pins into the femur. A 0.25 mm osteotomy was subsequently created using a 0.22 mm Gigli saw, which ensured an accurate and reproducible osteotomy width. One to two drops of a Lidocaine 1%/Bupivicaine 0.5% cocktail were then applied directly to the osteotomy site, providing both fast-acting and long-lasting analgesia. The skin was sutured using 6-0 Prolene.

### Post-surgical distraction procedure

By turning the threaded rod of the fixator system, the proximal and distal segments of the osteotomized femur could be distracted or compressed at a controlled rate. This allowed for full and non-invasive control over the osteotomy size post-operatively. We rotated the During a distraction or compression event, the fixator screw was rotated by 144°, which resulted in a 0.2 mm translation, with clockwise and counterclockwise rotations leading to distraction and compression, respectively (Fig. 2A). The femur was also subjected to some uncontrolled loading over the healing period, as mice were allowed to move and ambulate unrestricted.

Following the osteotomy procedure, all mice underwent a latency period of 5 days (Fig. 1). Groups 1, 3, and 4 then underwent a 10-day distraction phase consisting of two 0.2 mm distractions per day at 12-h intervals, for a total distraction gap of 4 mm. Meanwhile, Group 2 (distraction AT) was subjected to four manipulations per day separated by 6-h intervals during the distraction phase: two 0.2 mm distractions, followed by a 0.2 mm compression and, finally, another 0.2 mm distraction, for a total distraction gap size of 4 mm after the 10-day period. Following the distraction phase, Groups 1 and 2 were allowed to heal undisturbed during the 35-day consolidation phase, while Groups 3 and 4 underwent the accordion technique at days 15–25 and 25–35 post-osteotomy, respectively. The accordion technique consisted of two manipulations per day separated by 12-h intervals: a 0.2 mm compression in the morning, followed by a 0.2 mm distraction in the evening. A compression and distraction amplitude of 0.2 mm was selected in order to reach a final gap size in of 4 mm in 10 days, a distraction phase time scale in line with other DO animal studies^19,21^. The pattern of two manipulations per day, once in the morning and once in the evening, was also based on a previous clinical study which performed manipulations with similar timing^13^.

4 mice from Group 3 required euthanasia at day 45 due to swelling of the foot. A veterinarian was consulted, who hypothesized that these symptoms may have been the result of sciatic nerve damage or vascular injury resulting from the early consolidation AT procedure.

### In vivo microcomputed tomography (microCT)

In vivo microCT was conducted on day 5, 15, 25, 35, 45 and 50 and was used to assess the formation of mineralized callus tissue (calcified cartilage and bone) throughout the healing period. A total volume of interest (VOI) of 5 mm was analyzed—this included the 4 mm post-distraction osteotomy gap, as well as an additional 0.5 mm at the proximal and distal ends of the osteotomy. The VOI was analyzed using the manufacturer’s software (CTan, Bruker) to determine whether bridging had occurred as well as quantify the following bone healing parameters: Bone Volume (BV, mm^3^), Total Volume (TV, mm^3^) and Bone Volume Fraction (BV/TV, mm^3^/mm^3^).

The construction of the distractor device was such that the titanium threaded bar was located between the x-ray source and detector during image acquisition for in vivo microCT analysis. This fixator design resulted in some visible metal artifact during in vivo microCT scanning, and, as a result, we did not quantify bone mineral density (BMD) and bone mineral content (BMC). Nevertheless, image quality was sufficient for the analysis of volumetric parameters, as well as for use in the generation of our in silico model, to assess regeneration in the challenging healing scenario presented in our experiment.

### Histological assessment of healing

At the experimental endpoint on day 56 post-osteotomy, mice were anesthetized with inhaled isoflurane before being euthanized by CO2 inhalation and cervical dislocation. Following fixation and decalcification, dissected femurs were then embedded in paraffin and sliced to a thickness of 4 µm before being stained with Movat Pentachrome and imaged with a microscope (Bioquant) at 10× magnification. Quantitative histological analysis of callus tissue composition was performed using commercially available software (Bioquant, Tennessee, USA).

### Statistical analysis for in vivo studies

Bridging scores are presented as percentage of mice per group in which bridging of mineralized tissue was observed, while all other in vivo microCT and histological parameters are presented as mean ± standard deviation. Statistical analysis of bridging score was carried out using Fisher’s exact test (p < 0.05). For microCT outcomes, the effects of microCT time point (day 5, 15, 25, 35, 45, 50) and Treatment group (DO, early consolidation AT, late consolidation AT, distraction AT) were assessed by ANOVA and post hoc Tukey tests (SAS 9.4, Cary, NC, USA) with statistical significance set to p < 0.05. For callus tissue composition (both absolute and fractional tissue areas), the effect of treatment was assessed via ANOVA and independent t-tests.

### Finite element modeling of the distraction site

As described in detail in our recent study^26^, a 2D axisymmetric finite element model was created based on the microCT scans of the distraction site taken at day 5 post-osteotomy, before the beginning of the distraction phase (endosteal diameter: 1.2 mm, periosteal diameter: 1.6 mm, gap size: 0.3 mm) (Fig. 2D). The cortical bone, bone marrow and callus tissue were meshed with CAX6MPH elements (ABAQUS, v 6.14-1, Dessault Systèmes Simulia Corp, Providence, RI, USA). A 2D axisymmetric finite element model was used, as it is less computationally expensive and more efficient when dealing with distraction-related remeshing compared to an actual 3D model. All tissues were assumed to be linear poroelastic, with the elastic modulus of cortical bone approximating the low limit of the average value reported for the mouse femurs in the literature^22,33^. The fixator was modeled as a linear spring with the axial stiffness determined by finite element analysis of the bone-fixator construct^26^.

Loading of the finite element model was consistent with that of the animal experiment. During the consolidation phase, a compressive load (1.5 N), representing normal activities of the mouse^22^, was calculated based upon the average body weight of this group of mice and applied to the model^26^. A detailed description of the loading and boundary conditions is shown in Fig. 2D.

### Mechanobiological simulation of tissue differentiation

Tissue differentiation during the bone regeneration process of DO was simulated with a previously published two-stage (distraction and consolidation) mechano-regulatory model for DO of the mouse bone^26^. This model describes the patterns of tissue differentiation during distraction and consolidation affected by different mechanical stimuli during DO^30^. The mechano-regulatory model was implemented with a fuzzy logic-based technique. This procedure has been described in detail in our previous study^26^. Briefly, tissue differentiation was processed as an initial value problem based on two mechanical (dilatational (ε) and distortional (γ) strains in the mechano-regulatory model) and five biological state variables (blood perfusion, cartilage concentration and bone concentration, as well as blood perfusion and bone concentration in adjacent elements)^31,34–37^ (Supplementary Information).

## Supplementary Information

Supplementary Information.

## Data Availability

All data generated or analysed during this study are included in this published article (and its Supplementary Information files).
