# Supplementary material for: The accordion technique did not improve bone healing in a mouse model of distraction osteogenesis
Source: Sci Rep. 2024 Oct 18;14:24466. doi: 10.1038/s41598-024-71335-0 (PMC11489654; doi:10.1038/s41598-024-71335-0)
Supplement: Supplementary file 1 — Supplementary Information. [file 41598_2024_71335_MOESM1_ESM.docx]

## In Vivo Microcomputed Tomography (microCT)

In vivo microCT was conducted on day 5, 15, 25, 35, 45 and 50 and was used to assess the formation of mineralized callus tissue (calcified cartilage and bone) throughout the healing period.An isotropic voxel size of 8 µm (SkyScan1276, Bruker) was used along with the following source parameters: 70 kV, 114µA, 0.5 Al filter, 0.3^o^ rotation step for a 180^o^ scan. Mice were anesthetized using inhaled isoflurane (1.5%-2% pure isoflurane oxygen mixture) and placed in a custom 3D-printed mouse bed in the scanner, which enabled optimal positioning of the left femur for scanning. Images were reconstructed (Nrecon, Bruker) using standard reconstruction algorithms provided with the machine.

Callus tissue mineral density (BMD, mg HA/cm^3^) was not reported since the presence of the titanium distractor bar likely resulted in metal artifact influencing this outcome.

The 3D uCT images were also used to quantify the number of mice which achieved bony bridging. The percentage of combined periosteal and intracortical bridging was calculated as the number of mice in each experimental group that achieved mineralized tissue bridging divided by the total number of mice in each group.

A global threshold of 70% of the mineral density of an intact portion of the femur away from the osteotomy gap, equivalent to 544 mg HA/cm^3^ was calculated. This threshold was then used to segment mineralized callus tissue (bone and calcified cartilage) from nonmineralized tissue in the callus as well as background

## Histological assessment of healing

At 50 days post-osteotomy, the mice were euthanized via isoflurane and CO2 inhalation, followed by cervical dislocation. Femurs were dissected and fixed for 48 hours in 4% paraformaldehyde before being decalcified in 10% Ethylenediaminetetraacetic acid for 24 days.

Following fixation and decalcification, dissected femurs were then embedded in paraffin and sliced to a thickness of 4 µm before being stained with Movat Pentachrome and imaged with a microscope (Bioquant) at 10X magnification. Quantitative histological analysis of callus tissue composition was performed using commercially available software (Bioquant, Tennessee, USA). A single fixed region of interest (ROI) was employed to carry out callus tissue area analysis. Longitudinally, this ROI included the 4mm distraction gap, as well as 0.5mm on either side, for a total ROI length of 5mm. Using the Bioquant software, segmentation by tissue type was carried out for fibrous connective tissue, bone, bone marrow, and cartilage. Area (mm^2^) was quantified for each tissue type, and tissue fractions (%) were subsequently calculated based on the total callus area within the ROI.

**Mechanobiological simulation of tissue differentiation**

Tissue differentiation during the bone regeneration process of DO was simulated with a previously published two-stage (distraction and consolidation) mechano-regulatory model for DO of the mouse bone [1]. This model describes the patterns of tissue differentiation during distraction and consolidation affected by different mechanical stimuli during DO [2]. The mechano-regulatory model was implemented with a fuzzy logic-based technique. This procedure has been described in detail in our previous study [1]. Briefly, tissue differentiation was processed as an initial value problem based on two mechanical (dilatational (ε) and distortional (γ) strains in the mechano-regulatory model) and five biological state variables (blood perfusion, cartilage concentration and bone concentration, as well as blood perfusion and bone concentration in adjacent elements) [3-7].

The initial callus, consisting of avascular soft tissue (the hematoma), possessed no blood perfusion (0%). Bone marrow and cortical fragments were rich with blood vessels and had blood perfusion of 100% [10]. All seven state variables were used to predict tissue differentiation throughout the processes of angiogenesis, endochondral ossification, chondrogenesis, cartilage calcification, and tissue destruction in the callus with a linguistic rule based fuzzy logic (Fuzzy Logic Toolbox in MATLAB, The MathWorks, Inc., Natick, MA, USA). According to the rules of tissue differentiation, fuzzy logic controller judged the input state (seven state variables) of each element in the callus area, and finally output the change of blood perfusion, cartilage and bone concentration to predict the result of tissue differentiation [3-7]. Following each step of tissue differentiation, the biological state variables of each callus element were updated:

$\text{c}_{\text{i+1. tiss}}\text{=}\text{∆}\text{c}_{\text{i. tiss}}\text{∆}\text{t}\text{+}\text{c}_{\text{i. tiss}}$ (1)

where $\Delta\text{t}$ is the time step, $\text{c}_{\text{i+1. tiss}}$ and $\text{c}_{\text{i. tiss}}$ are the current time step and the previous time step tissue concentration for each type of tissues in the callus element, respectively. The material properties of the callus area were then updated by using mixture rules according to the current biological state variables [10]. The Young’s modulus *E* was calculated by multiplying the tissue modulus by the third power of the tissue concentration:

*E*=$\sum_{\text{tiss}} \text{E}_{\text{tiss}}\text{c}_{\text{tiss}}^{\text{3}}$ (2)

where $\text{E}_{\text{tiss}}$ and $\text{c}_{\text{tiss}}$ are the current time step tissue modulus and tissue concentration in the callus element, respectively. Others used a linear rule of mixture, such as Poisson's ratio *ν*:

*ν*=$\sum_{\text{tiss}} \nu_{\text{tiss}}\text{c}_{\text{tiss}}$ (3)

where $\nu_{\text{tiss}}$ is the current time step tissue Poisson's ratio in the callus element. Our previous study verified the accuracy of the simulation algorithm by comparing model-predicted bone formation with the experimental results from mice undergoing conventional DO [1].

## References

1. Fu, R., et al., *In vivo and in silico monitoring bone regeneration during distraction osteogenesis of the mouse femur.* Comput Methods Programs Biomed, 2022. **216**: p. 106679.

2. Fu, R., et al., *Mechanical regulation of bone regeneration during distraction osteogenesis.* Medicine in Novel Technology and Devices, 2021. **11**: p. 100077.

3. Niemeyer, F., et al., *Simulating lateral distraction osteogenesis.* PLoS One, 2018. **13**(3): p. e0194500.

4. Fu, R., et al., *The combined effects of dynamization time and degree on bone healing.* J Orthop Res, 2022. **40**(3): p. 634-643.

5. Shefelbine, S.J., et al., *Trabecular bone fracture healing simulation with finite element analysis and fuzzy logic.* J Biomech, 2005. **38**(12): p. 2440-50.

6. Simon, U., et al., *A numerical model of the fracture healing process that describes tissue development and revascularisation.* Comput Methods Biomech Biomed Engin, 2011. **14**(1): p. 79-93.

7. Fu, R., et al., *Enhancing the Efficiency of Distraction Osteogenesis through Rate-Varying Distraction: A Computational Study.* Int J Mol Sci, 2021. **22**(21).
